# Membrane Transport Proteins Expressed in the Renal Tubular Epithelial Cells of Seawater and Freshwater Teleost Fishes

**DOI:** 10.3389/fphys.2022.939114

**Published:** 2022-06-23

**Authors:** Akira Kato, Ayumi Nagashima, Kohei Hosono, Michael F. Romero

**Affiliations:** ^1^ School of Life Science and Technology, Tokyo Institute of Technology, Yokohama, Japan; ^2^ Department of Physiology and Biomedical Engineering, Nephrology and Hypertension and O’Brien Urology Research Center, Mayo Clinic College of Medicine & Science, Rochester, MN, United States

**Keywords:** fish physiology, seawater acclimation, freshwater acclimation, renal tubule, membrane transport protein, osmoregulation, sulfate homeostasis, magnesium homeostasis

## Abstract

The kidney is an important organ that maintains body fluid homeostasis in seawater and freshwater teleost fishes. Seawater teleosts excrete sulfate and magnesium in small amounts of isotonic urine, and freshwater teleosts excrete water in large amounts of hypo-osmotic urine. The volume, osmolality, and ionic compositions of the urine are regulated mainly by membrane transport proteins expressed in the renal tubular epithelial cells. Gene expression, immunohistochemical, and functional analyses of the fish kidney identified membrane transport proteins involved in the secretion of sulfate and magnesium ions by the proximal tubules and reduction of urine volume by the collecting ducts in seawater teleosts, and excretion of water as hypotonic urine by the distal tubules and collecting ducts in freshwater teleosts. These studies promote an understanding of how the kidney contributes to the seawater and freshwater acclimation of teleosts at the molecular level.

## 1 Introduction

**FIGURE 1:**
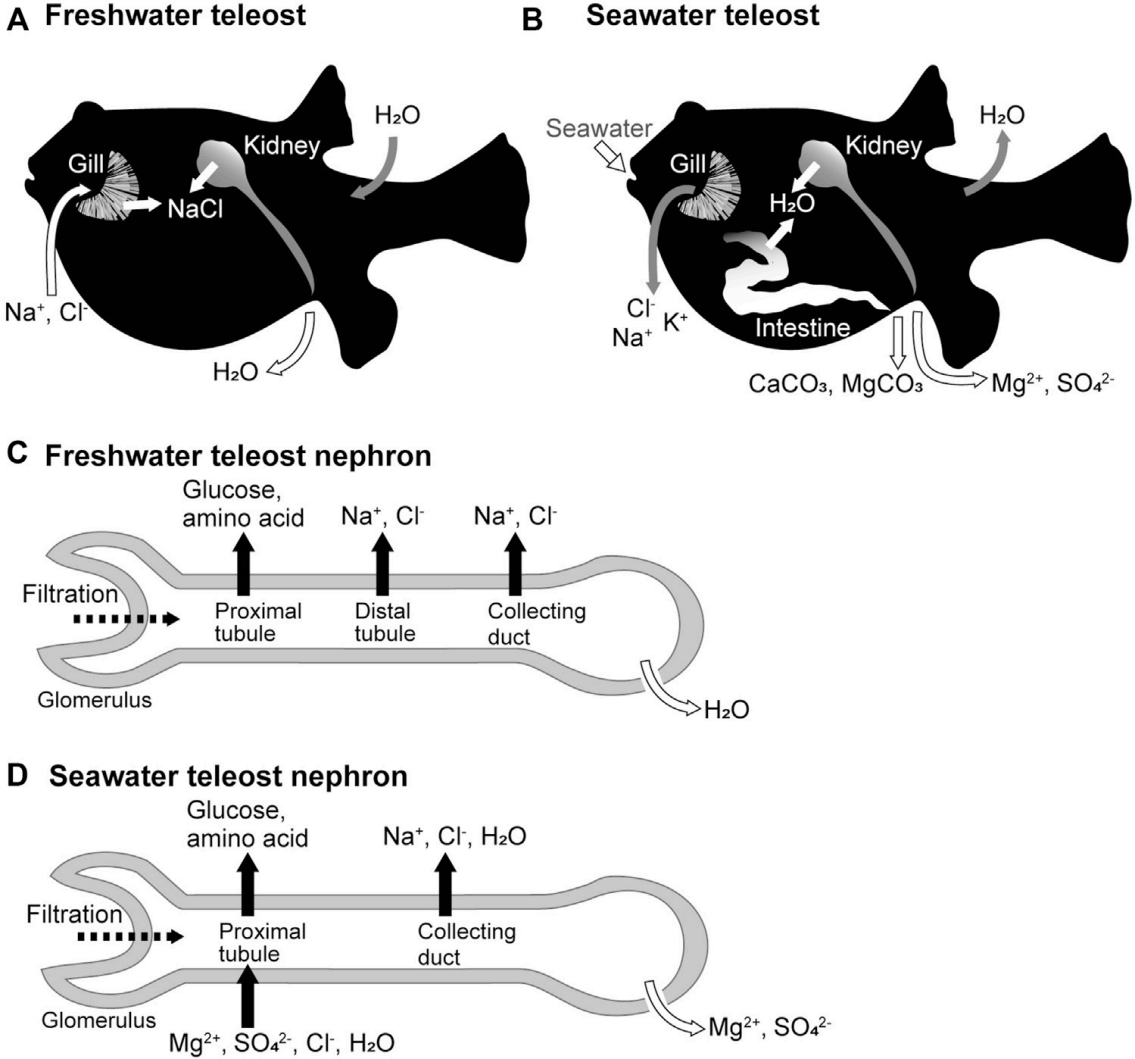
Freshwater and seawater acclimations of teleost fishes and organ functions. **(A,B)** Movement of ions and water across the epithelia of the gill, kidney, and intestine of freshwater and seawater teleosts. **(C,D)** Ion and water movement in the nephrons of freshwater and seawater teleosts.

The ionic compositions and osmolarity of body fluids in seawater and freshwater teleost fishes are similar to those in humans and other mammals. Freshwater teleosts live in environments with considerably lower osmolarity than their body fluids. To balance water entry and loss of salts in freshwater environments, the kidneys actively produce a hypotonic urine with salt concentrations that is 1/10–1/20 of that of the body fluid, and the branchial ionocytes (Hwang et al., 2011), mitochondrion-rich cells scattered along the lamellae, and intestinal epithelia absorb salts from the environmental water and food, respectively (Figure 1A). Conversely, seawater teleosts live in environments that have approximately three-fold higher osmolarity than their body fluids. To balance salt entry and loss of water in seawater environments, seawater is ingested, salt and water are absorbed from the ingested seawater via the intestinal epithelial cells, Cl^-^, Na^+^, and K^+^ are excreted from the branchial ionocytes, and Mg^2+^ and SO_4_^2-^ are excreted in the isotonic urine (Figure 1B). The mechanism of body fluid homeostasis in freshwater and seawater teleosts can be understood as the balance of the epithelial transport of ions and water by the branchial ionotyes, renal tubules, and intestinal epithelial cells. The molecular mechanisms of these processes can be explained by the membrane transport proteins (channels, transporters, and pumps) expressed in the plasma membrane of these epithelial cells. Here, we focus on the functional differences in the kidney of seawater and freshwater teleosts, and review the membrane transport proteins that are expressed in the renal tubular epithelial cells of teleost fishes.

## 2 Functional Differences in the Kidneys of Freshwater and Seawater Teleosts

The kidney is the only organ in vertebrates that can produce urine (Schmidt-Nielsen, 1997). Urine is produced by blood filtration, followed by ion and water secretion and reabsorption by nephrons. The glomeruli are responsible for filtration, while the tubules are responsible for secretion and reabsorption. Urine volume is controlled balancing the amount of water filtered by the glomerulus and the amount of water secreted and reabsorbed by the tubules. The urine composition is also regulated by the secretion and reabsorption of selective ions by the tubules.

In freshwater fish, the kidney functions as the organ responsible for water excretion (Hickman and Trump, 1969; Marshall and Grosell, 2006). The glomeruli of freshwater teleosts filter more blood and produce more primary urine than those of seawater teleosts (Fleming and Stanley, 1965; Nishimura and Imai, 1982). The proximal tubules, which are the renal tubule segments closest to the glomerulus, reabsorb nutrients such as glucose and amino acids from the primary urine (Dickman and Renfro, 1986). The distal tubules and collecting ducts actively reabsorb Na^+^ and Cl^-^ from the primary urine and are called diluting segments because they do not permeate much water. As a result, the freshwater fish kidney produces hypotonic urine resulting in net water excretion (Nishimura et al., 1983) (Figure 1C).

In seawater fish, the kidney functions as an organ responsible for the excretion of divalent ions (Mg^2+^, SO_4_^2-^, and so on) (Hickman and Trump, 1969; Marshall and Grosell, 2006). The proximal tubules of seawater fish actively secrete fluid containing Mg^2+^, SO_4_^2-^, and Cl^-^ into the tubular lumen (forming urine), a function not observed in the kidneys of freshwater fish or terrestrial animals (Beyenbach et al., 1986; Beyenbach, 2004). The collecting ducts may then actively reabsorb water along with Na^+^ and Cl^-^ to reduce the urine volume, producing a relatively small volume of isotonic urine with high concentrations of Mg^2+^ and SO_4_^2-^ (Figure 1D).

## 3 Molecular Mechanisms Underlying Reabsorption and Secretion by the Renal Tubular Cells

**FIGURE 2:**
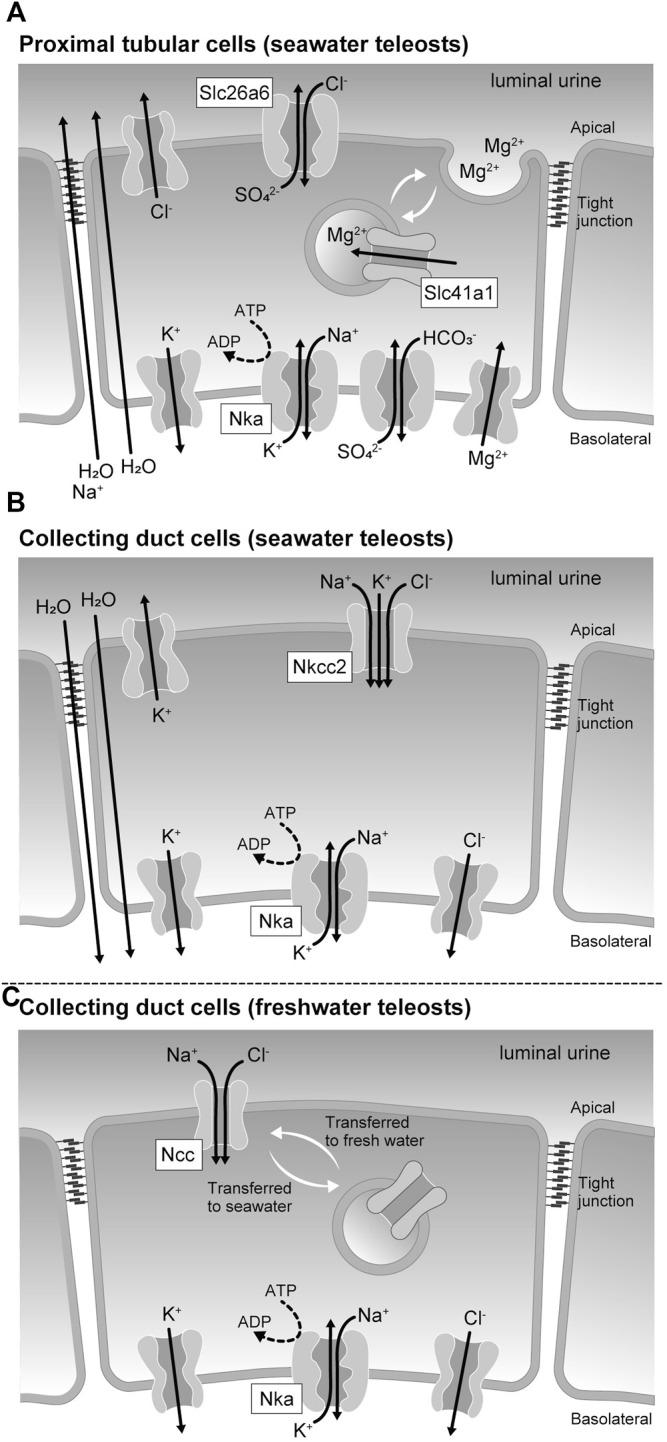
Membrane transport proteins expressed in the renal tubular epithelial cells of seawater and freshwater teleost fishes. **(A)** Membrane transporters involved in the sulfate and magnesium ion secretion mechanism by proximal tubular cells of seawater teleosts. **(B)** Membrane transporters involved in the urinary volume reduction mechanism by the collecting duct cells of seawater teleosts. **(C)** Membrane transporters responsible for the urine dilution by the collecting duct cells of freshwater teleosts.

Reabsorption and secretion in the renal tubules are mediated by a single layer of renal tubular epithelial cells. Cells are directly connected to each other by cell-cell adhesions, including tight junctions, which function as barriers separating the primary urine from the tissue fluid. Cell-cell adhesions are also responsible for transporting various substances during renal tubular reabsorption and secretion, and the tight junction protein claudins regulate the paracellular permeability (Tsukita et al., 2019). The tight junction also separates the apical and basolateral membrane domains in epithelial cells (Figure 2). Epithelial transport can occur through the transcellular pathway via the apical membrane, cytoplasm, and basolateral membrane of epithelial cells and through the paracellular pathway via the intercellular spaces between epithelial cells. The basolateral membrane of tubular cells, containing sodium pumps (Nkas, Na^+^/K^+^-ATPases), potassium channels, and chloride channels, maintains a low Na^+^, low Cl^-^, and high K^+^ intracellular environment (Figure 2) and generates inside negative membrane potentials. The high Na^+^ and Cl^-^ contents of the extracellular fluids create an ionic gradient between the inside and the outside of the cell. These chemical gradient and membrane potentials (electrical gradients) are used as driving forces for secondary active transport through various cotransporters and exchangers.

The use of fish genome data has drastically accelerated the research in identifying membrane transport proteins in the renal tubular epithelial cells responsible for seawater and freshwater acclimation of fishes. The genomes of many fish species have recently been sequenced by the development of next-generation sequencers. However, we have focused on two closely related fish species, the euryhaline species river pufferfish (*Takifugu obscurus*) and the marine species Japanese pufferfish (*Takifugu rubripes*) (Kato et al., 2005) because the whole genome data of Japanese pufferfish was published in 2002 (Aparicio et al., 2002). Comparing the expression levels of membrane transport protein families in the kidney of seawater-, brackish water-, and freshwater-acclimated *Takifugu* species identified some of the molecular mechanisms involved in seawater and freshwater acclimation of teleosts as described below.

## 4 Membrane Transport Proteins Involved in the Divalent Ion Secretion by the Proximal Tubular Cells of Seawater Teleosts

The solute carrier (Slc) 26 is a family of proteins with anion-exchange export activity (Mount and Romero, 2004; Alper and Sharma, 2013). Slc26 is mainly found in the plasma membrane and mediates the influx of Cl^-^ and efflux of various anions, such as HCO_3_^−^, SO_4_^2-^, and oxalate^2-^. A member of this family, Slc26a6, is expressed in the kidney and intestine of river pufferfish and the Japanese eel (*Anguilla japonica*), and its expression increases during seawater acclimation (Kato et al., 2009; Watanabe and Takei, 2011). Electrophysiological analysis of pufferfish Slc26a6 expressed in *Xenopus laevis* oocytes showed that it has a very highly electrogenic Cl^-^/SO_4_^2-^ exchange activity. In the kidney of seawater-acclimated river pufferfish and Japanese eel, Slc26a6 localizes to the brush border of the apical membrane of the proximal tubule. These results are consistent with studies that showed the Cl^-^/SO_4_^2-^ exchange activity of brush border membrane vesicles isolated from the kidney of the seawater teleost southern flounder (*Paralichthys lethostigma*) (Renfro and Pritchard, 1983), and suggest that Slc26a6 is at least one of the major pathways of apical SO_4_^2-^ secretion in the proximal tubule of seawater teleosts (Figure 2A). Slc26a1, another SO_4_^2-^ transporter of the Slc26 family, localizes to the basolateral membrane of the proximal tubule in the eel kidney (Nakada et al., 2005; Watanabe and Takei, 2011). When expressed in *Xenopus* oocytes, fish Slc26a1 shows robust SO_4_^2-^ transport activity (Nakada et al., 2005), but the mode of this transport activity has not yet been established. Analysis of human Slc26a1 expressed in *Xenopus* oocytes indicated that Slc26a1 has Cl^-^-dependent pH-sensitive HCO_3_^−^/SO_4_^2-^ exchange activity (Wu et al., 2016). These results suggest that Slc26a1 mediates basolateral SO_4_^2-^ uptake for luminal SO_4_^2-^ secretion (Figure 2A).

Renal SO_4_^2-^ excretion has also been observed in seawater elasmobranch species. In elephant fish (*Callorhinchus milii*), Slc26a6 and Slc26a1 are expressed in the apical and basolateral membranes, respectively, of the renal proximal tubule II segments and exhibit SO_4_^2-^ transport activity when expressed in *Xenopus* oocytes (Hasegawa et al., 2016). These results suggest that the membrane transport proteins involved in renal tubular SO_4_^2-^ secretion are conserved between seawater elasmobranches and teleosts.

A family of proteins homologous to the bacterial Mg^2+^ transporter MgtE was found in vertebrates and named the Slc41 (Sahni and Scharenberg, 2013). Slc41a1 is expressed in the pufferfish kidney, and its expression levels increase during seawater acclimation (Islam et al., 2013). Increased salinity also stimulates the renal expression of Slc41a1 in the euryhaline glomerular fish Atlantic salmon (*Salmo salar*) and the aglomerular marine gulf toadfish (*Opsanus beta*) (Madsen et al., 2020; Hansen et al., 2021). While the activity of fish Slc41a1 has not been successfully determined, human Slc41a1 has been reported to possess Na^+^/Mg^2+^ exchange activity (Kolisek et al., 2012). Immunohistochemical analysis at the light and electron microscopic levels showed that Slc41a1 was localized to vacuoles in the apical cytoplasm of the proximal tubules in seawater-acclimated river pufferfish (Islam et al., 2013). The proximal tubules of seawater fish kidneys have intracellular granules containing high Mg^2+^ concentrations and are the site of active secretion of Mg^2+^ into the lumen (Chandra et al., 1997; Beyenbach, 2000). These results suggest that Slc41a1 is involved in a pathway that concentrates Mg^2+^ in intracellular granules that are then secreted into the luminal fluid by exocytosis (Figure 2A).

Another family of proteins that are homologous to the bacterial magnesium transporter CorC was found in vertebrates and named Cnnm (cyclin M or cyclin and CBS domain divalent metal cation transport mediator) family (Funato and Miki, 2019). Again, the activity of fish Cnnm has not been successfully analyzed, however, mammalian Cnnm is known to mediate plasma membrane Mg^2+^ efflux (Hirata et al., 2014). In the kidneys of river pufferfish, the expression of Cnnm3 is upregulated in seawater, while that of Cnnm2 is increased in freshwater, and Cnnm3 is expressed in the lateral membrane of proximal tubular cells (Islam et al., 2014). The role of Cnnm3 in Mg^2+^ secretion remains unclear, however, these results suggest that Cnnm3 is involved in paracellular Mg^2+^ secretion.

## 5 Membrane Transport Proteins Involved in Divalent Ion Concentration and Volume Reduction of Urine by the Collecting Duct Cells of Seawater Teleosts

In the kidney of seawater teleosts, the renal collecting ducts and urinary bladder reabsorb Na^+^, Cl^-^, and water to reduce the volume of urine. As a result, Mg^2+^ and SO_4_^2-^ are highly concentrated in the iso-osmotic urine, which is finally excreted from the urinary bladder (Hickman and Trump, 1969; Marshall and Grosell, 2006). Slc12a1, also called Na^+^/K^+^/2Cl^-^ cotransporter 2 (Nkcc2), is highly expressed in the collecting ducts of marine pufferfish (Kato et al., 2011). In the mammalian kidney, Nkcc2 and K^+^ recycling channels in the apical membrane mediate Na^+^ and Cl^-^ reabsorption (Arroyo et al., 2013). In the collecting ducts of marine pufferfish, Nkcc2 may promote the reabsorption of Na^+^ and Cl^-^, which could be a driving force for water reabsorption and urinary volume reduction (Figure 2B).

## 6 Membrane Transport Proteins in the Distal Tubule and Collecting Duct Cells of Freshwater Teleosts Involved in Urine Dilution

In general, both freshwater and euryhaline teleosts that can live in freshwater have freshwater fish type nephrons (Hickman and Trump, 1969; Kato et al., 2011). In the kidneys of freshwater teleosts and freshwater-acclimated euryhaline teleosts, the distal tubules and collecting ducts reabsorb Na^+^ and Cl^-^, but not water, thereby producing a large amount of hypotonic urine. Therefore, these tubules act as diluting segments in the kidney of teleosts. Both Slc12a1 (Nkcc2) and Slc12a3 (Ncc, Na^+^/Cl^-^ cotransporter) are highly expressed in the kidneys of freshwater and euryhaline fishes (Katoh et al., 2008; Kato et al., 2011). This is in contrast to the stenohaline seawater fish that expresses Nkcc2 but does not or scarcely expresses Ncc in the kidney (Kato et al., 2011). Nkcc2 and Ncc are localized in the distal tubules and collecting ducts, respectively. In freshwater pufferfish collecting ducts, Ncc is localized to the luminal side of the plasma membrane, whereas in seawater pufferfish collecting ducts, Ncc on the plasma membrane is incorporated into intracellular granules and is downregulated (Figure 2C). These data suggest that Ncc-mediated Na^+^ and Cl^-^ reabsorption is particularly important for freshwater acclimation of teleosts.

## 7 Conclusion

The proximal tubule is an important secretory pathway for divalent ions in seawater teleosts. The proximal tubules of seawater fish kidneys secrete SO_4_^2-^ directly into the lumen via membrane transporters on the plasma membrane, whereas Mg^2+^ is stored in intracellular granules and excreted into the lumen. It is interesting to understand why these differences in secretion mechanisms are necessary. The collecting duct is important for freshwater teleosts as the site of water elimination (production of hypotonic urine) and is important for seawater teleosts as a site of urine volume reduction. This difference can be explained by the differential water permeability of the collecting ducts between seawater and freshwater teleosts, and the mechanisms controlling these differences are expected to be elucidated in the future. Na^+^ and Cl^-^ reabsorptive activity of the distal nephrons is important in both seawater and freshwater teleosts, but freshwater fish utilize both Nkcc2 and Ncc whereas seawater fish utilize only Nkcc2. Further studies will be needed to elucidate why Ncc is necessary for the functioning of the kidneys of freshwater teleosts.
